# Deep learning-based recommendation system for metal–organic frameworks (MOFs)†

**DOI:** 10.1039/d4dd00116h

**Published:** 2024-06-10

**Authors:** Xiaoqi Zhang, Kevin Maik Jablonka, Berend Smit

**Affiliations:** a Laboratory of Molecular Simulation (LSMO), Institut des Sciences et Ingénierie Chimiques, Ecole Polytechnique Fédérale de Lausanne(EPFL) Rue de l'Industrie 17 CH-1951 Sion Valais Switzerland berend.smit@epfl.ch; b Laboratory of Organic and Macromolecular Chemistry (IOMC), Friedrich Schiller University Jena Humboldtstrasse 10 07743 Jena Germany; c Helmholtz Institute for Polymers in Energy Applications Jena (HIPOLE Jena) Lessingstrasse 12-14 07743 Jena Germany

## Abstract

This work presents a recommendation system for metal–organic frameworks (MOFs) inspired by online content platforms. By leveraging the unsupervised Doc2Vec model trained on document-structured intrinsic MOF characteristics, the model embeds MOFs into a high-dimensional chemical space and suggests a pool of promising materials for specific applications based on user-endorsed MOFs with similarity analysis. This proposed approach significantly reduces the need for exhaustive labeling of every material in the database, focusing instead on a select fraction for in-depth investigation. Ranging from methane storage and carbon capture to quantum properties, this study illustrates the system's adaptability to various applications.

## Abstract 2

This work presents a recommendation system for metal–organic frameworks (MOFs) inspired by online content platforms.

## Graphical abstract

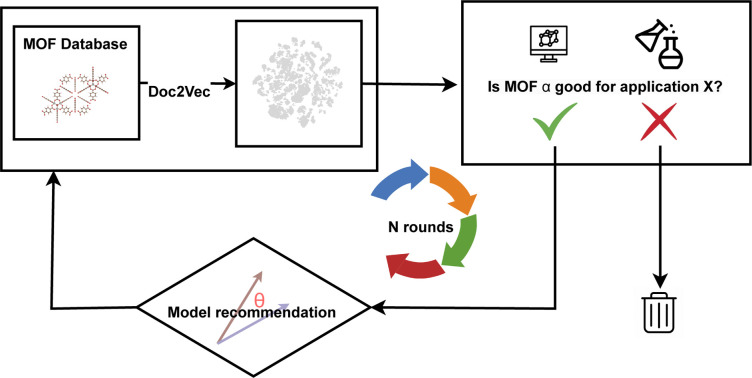

## 1 Introduction

Metal–organic frameworks (MOFs) are crystalline materials composed of metal ions or clusters connected by organic linkers. MOFs' unique structural characteristics, tunability, and high porosity have attracted significant attention in various research and industrial applications such as gas storage and separation, catalysis, sensing, and drug delivery.^1,2^ With the ever-growing number of synthesized MOFs^3,4^ and the increasing number of applications,^5,6^ it is time-consuming and labor-intensive to measure the key performance indicators (KPIs) for each material and each specific purpose.^7^

In recent years, considerable efforts have been made to address this challenge using high-throughput simulation and machine learning (ML).^8–14^ Rosen *et al.*^15^ introduced the QMOF database and showed the power of machine learning in discovering MOFs with targeted electronic structure properties. In the gas-related field, there are various high-throughput screenings and ML models designed for Xe/Kr separation,^16,17^ hydrogen storage,^18,19^ carbon dioxide capture,^20–22^ to name a few. Although these efforts provide valuable data and resources to the MOF community, developing these supervised machine-learning models relies on large-scale computation or experiments. Besides, researchers cannot always find a developed ML model that perfectly matches their needs.

To reduce the cost of labeling a database for a specific application from scratch, more and more trials focus on leveraging pre-trained models and transferring them to related tasks. It reduces data requirements while preserving model efficacy.^23,24^ For example, Ma *et al.*^25^ applied transfer learning from a source model trained for H_2_ adsorption at specific conditions to predict H_2_ adsorption under varied conditions and different gas species. Lim and Kim^26^ showcased knowledge from methane adsorption properties can enhance predictions of methane diffusion properties within MOFs. Despite these successful examples, the performance of transfer learning considerably depends on the task similarity. Methods like Bayesian optimization^27^ and genetic algorithm^28^ have been proposed to reduce computational expense. However, these methods require either well-designed acquisition functions or extensive tuning and iterations. Bearing this in mind, we would like a universal tool for MOFs that requires minimal labeling effort for development and is easily scalable to newly designed MOF databases.

Inspired by online recommendation platforms for movies or articles, we aim to develop a recommendation system for MOFs. Conceptually, this system functions similarly to online recommenders; it generates a pool of interesting materials for specific applications based on user-endorsed MOFs. The model learns MOF embedding vectors without supervision, suggesting materials by assessing their similarities to the known top-performing structures. This approach eliminates the need for exhaustive labeling of every material in the database, focusing instead on a fraction for in-depth investigation. Sturluson *et al.*^29^ were among the first to link recommenders and properties of nanoporous materials and used this analogy as inspiration for dealing with materials for which a gas adsorption property was missing. In this work, we use the analogy directly: recommend similar materials guided by candidates for a specific application.

Our recommendation system harnesses the power of the Doc2Vec model, a task-agnostic and data-driven representation learning approach for document-structured data.^30^ It adapts to various applications similarly to how a movie or document recommender tailors suggestions to user preferences. The comprehensive characteristics of MOFs, including the crystallographic information, geometric descriptors, and topology, in the document-structured data ensures its ability to capture gas adsorption, separation, and quantum properties. When faced with an application or a MOF database lacking prior knowledge, the model efficiently navigates researchers to a subset of structures with a minimal number of measurements. This is especially essential in the scenario where the measurements are expensive or laborious.

## 2 Recommendation model

### 2.1 Architecture

**Fig. 1:**
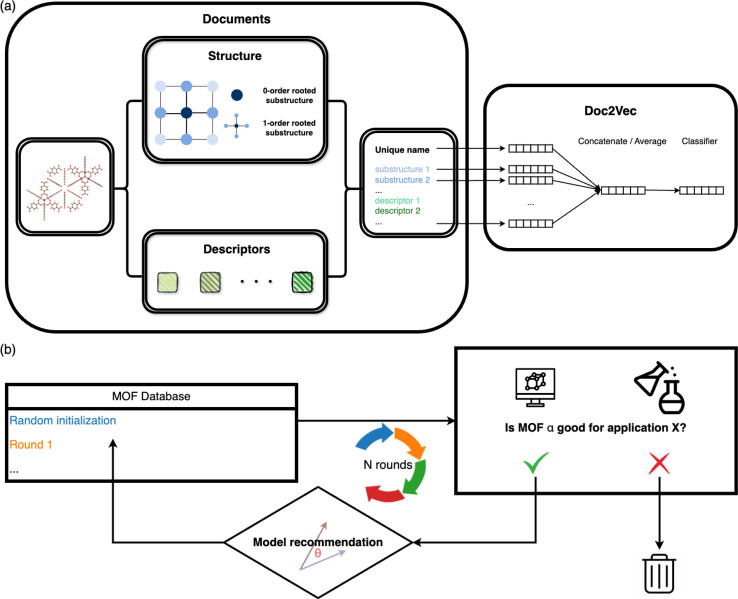
The recommendation model architecture is based on (a) Doc2Vec and (b) similarity analysis. (a) MOFs are encoded into document-like structures, encompassing inherent structure connectivities, geometric descriptors, and topology. These MOF documents are then input into the Doc2Vec model to obtain fixed-length name vectors and word vectors that can be used for similarity comparison in the subsequent recommendation process. (b) Overview of the iterative recommendation scheme: in each round, (i) simulations are used to evaluate the selected MOF subsets to determine their suitability for a specific application; (ii) the model subsequently suggests structures similar to the identified candidates within the database.

The overall architecture of the MOF recommendation system is based on the Doc2Vec algorithm.^30^ The methodology requires converting MOFs into document-like structures.^31–33^ The MOF documents comprise inherent structure connectivities and geometric descriptors, as depicted in the left panel of Fig. 1a. For each atom within a MOF, rooted substructures are identified and represented using ordered element symbols. Substructure searches are limited to the second-order neighbors to balance the need for distinct structures without overwhelming uniqueness. Beyond crystallographic information, the MOF documents incorporate geometric properties computed by Zeo++.^34^ Continuous values such as density and pore diameters are discretized into binned categories. Additionally, topology, often represented with three letters by reticular chemists that describe the arrangement and connectivity of MOF building blocks,^35^ is appended to the MOF documents to enrich the depiction of MOFs.

These document corpora are then fed into the unsupervised Doc2Vec model. We used the distributed memory algorithm when training the Doc2Vec model, as shown in the right panel of Fig. 1a. In this training process, the algorithm learns to associate words with document contexts, generating unique numeric vector representations for documents and words. We assigned a unique name to each MOF in the dataset to distinguish them, like the title of a document. Each MOF name is linked to a name vector, and every word in the document is associated with a fixed-length word vector. The numeric name vectors and word vectors are initialized to the same length. Throughout the training, contexts are sampled from sliding windows. Each name vector is shared across all the contexts sampled from one document. The name vector represents the missing information from the current context and can act as a memory of the overall MOF characteristics. The model endeavors to predict the next word in a context from averaged or concatenated name vector and word vectors. The name vector and word vectors are adjusted iteratively through this training process, ultimately capturing the semantic information shared across the document contexts.

### 2.2 Iterative recommendations based on similarity analysis

The underlying assumption guiding the recommendation is that MOFs with similar structural characteristics likely exhibit analogous performance in the same application. Our recommendation model generates fixed-length continuous vectors for document titles (MOF names) and words. The Doc2Vec model learns word semantics by capturing the contextual information surrounding each word. Through this process, words often occurring in similar contexts tend to have similar semantics, thereby being mapped closely in the embedding space.^36,37^ In parallel, document vectors, or name vectors as we term them, are learned by aggregating the context of all words within each document. This approach results in documents with akin contexts, *i.e.*, similar compositions, geometric properties, and topologies in the case of MOFs, being positioned proximally within the embedding space. Consequently, these distributed embedding vectors enable us to effectively measure the similarities or dissimilarities between the MOFs or words. The cosine similarity metric is employed for this purpose, calculated by1
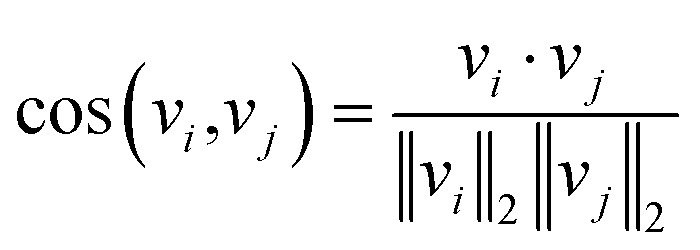
Here, *v*_*i*_ and *v*_*j*_ represent the vectors of MOF names or words we wish to evaluate. The similarity score ranges from −1 to 1.

In practical applications, we initiate the recommendation process with a subset of MOFs randomly selected from the entire database. Molecular simulations (for gas adsorption and separation) or density functional theory computations (for electronic properties) are then conducted to assess the performance of these chosen MOFs, ranking them based on KPIs specific to the relevant applications. In cases where multiple KPIs are considered, we calculate the summation of the ranks of all the KPIs. Highly ranked MOFs are subsequently queried to the model for the next rounds of selections. The model computes the cosine similarities of MOFs in the database with each queried MOF. The most similar ones are then returned. We combine all the returned structures with high similarity scores and filter the unique ones. Statistical analysis of the similarity scores of the MOF databases we used in this study can be found in Section S1.† The suggested structures are subsequently evaluated by simulations and again input into the model for the next recommendation round. We iteratively perform this process until the properties of suggested MOFs do not improve much. The overall scheme is illustrated in Fig. 1b.

## 3 Results

Researchers often seek or design new structures with analogous functionalities when a novel structure is discovered for a specific application, typically achieved through functionalization.^38,39^ Our model proves useful in populating the chemical landscape of interest by discerning the similarities between MOFs in the database. Among all the reported MOF databases,^15,40–42^ the ARC-MOF database^43^ contains both experimental and hypothetical structures, which is a good starting point for studying MOF application in gas adsorption and separation. To refine the original dataset and eliminate redundancies, we leverage the ARC-MOF database sourced from mofdscribe.^44^ In the following sections, we illustrate the practical use of our recommendation model in identifying a subset of compelling structures, especially when limited information about the materials in the database is available. This is demonstrated through two exemplary applications: methane storage and carbon dioxide capture.

Remarkably, the recommendation model performs well beyond gas adsorption and separation. We show its further application by suggesting MOFs with band gaps falling within specified ranges from the QMOF database,^15^ which is a fundamental property of interest for MOF application in gas sensing and detection,^45,46^ photocatalysis,^47,48^*etc.* This reveals the model's ability to capture MOF quantum characteristics.

### 3.1 Methane storage

Many research groups have focused on finding the optimal MOF for methane storage to promote using methane as an alternative energy source.^49,50^ In evaluating the methane storage capacity of porous materials, assessing their adsorption capacity is the fundamental step. The CH_4_ Henry coefficient, which reflects the affinity between gas and the framework, can serve as a proxy for methane adsorption capacity.^51^ An adsorbed natural gas (ANG) system consists of porous materials packed into a vessel to store methane at ambient pressure, where understanding methane storage at infinite dilution is crucial.^52^ Besides the adsorption capacity, optimal materials should also have a high deliverable capacity. The deliverable capacity is defined as the maximum amount of gas that can be released and quantified by the difference in methane loading between high pressure and low pressure.^53,54^ The CH_4_ Henry coefficient and deliverable capacity are considered as the key performance indicators (KPIs) in this study. We used molecular simulations to compute these KPIs for each material. The simulation details are in Section 6.1.

**Fig. 2:**
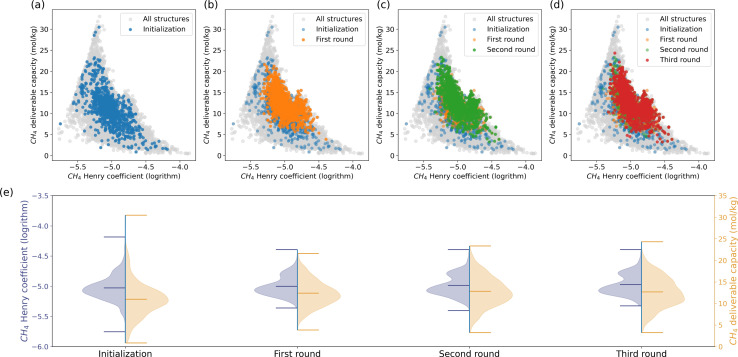
Material performance of (a) initialization and (b)–(d) three rounds of recommended structures in methane storage. 1000 structures are recommended in each round among around 22 000 MOFs in the ARC-MOF dataset. The (negatively correlated) CH_4_ Henry coefficient and deliverable capacity are considered in this case. The recommended structures gradually move towards the Pareto front across each round. The minimum, mean, and maximum KPIs of structures in each round are shown in (e). Considering the two competitive KPIs meanwhile, the model recommendations' maximum KPIs did not surpass the initialization. The model recommendations boast the minimum and mean KPIs.

We launched a recommendation process introduced in Section 2.2 to tackle this challenge. The process involves 1000 randomly selected structures in the initialization, spanning three iterative recommendation rounds as shown in the first row of Fig. 2. By ranking the simulated CH_4_ Henry coefficients and deliverable capacities, the top-performing structures are then queried to the model for another set of 1000 MOFs in the database with the highest similarity scores. Repetitive structures may be returned. We repeated the labeling process and model recommendations and stopped the iteration where the statistics of the KPIs of the recommended MOFs showed insignificant improvement compared to the last round.

Fig. 2e depicts the minimum, mean, and maximum KPIs for the structures in each round. The initialization structures are a good representative of the dataset. The model recommendations narrow down the ranges of the KPIs as a result of the competitive interplay between the CH_4_ Henry coefficient and deliverable capacity. The average Henry coefficient and deliverable capacity exhibit a steady increase across each round. Structures with CH_4_ Henry coefficient around 17.8 mol kg^−1^ MPa^−1^ boast a lot. Due to the competitive relationship between CH_4_ Henry coefficient and deliverable capacity, the three structures with the highest deliverable capacity in the initialization were abandoned in the recommendation stage. Instead, the model recommendations explored a lot of structures with deliverable capacity from 15 to 25 mol kg^−1^. The maximum deliverable capacity increases across the recommendation rounds.

### 3.2 Carbon capture

In a carbon capture process, we would like to separate CO_2_ from the flue gasses, of which the primary component is N_2_.^55^ The ideal material should have high CO_2_ selectivity and maximum CO_2_ recovered after an adsorption–desorption cycle.^50^ Therefore, we focus on two KPIs : CO_2_ Henry selectivity over N_2_ and CO_2_ working capacity. Unlike methane storage, these two KPIs exhibit a positive correlation. We followed the same procedure as the methane storage case, *i.e.*, assessing a subset of randomly selected 1000 MOFs by molecular simulation and querying the recommendation model for similar MOFs to the top-performing candidates iteratively.

**Fig. 3:**
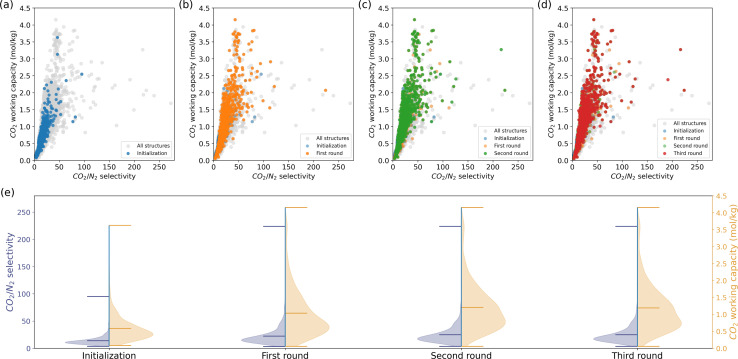
Iterative model recommendations for carbon capture, with a focus on two KPIs : CO_2_/N_2_ selectivity and CO_2_ working capacity. (a)–(d) illustrate the material properties of the initialization and recommendation subsets, with more and more structures with high KPIs highlighted across the rounds. Grey points denote all structures in the MOF database. (e) The distributions of KPIs for each round. The peak and tail of the distributions shift towards higher values across the recommendation rounds.

The outcomes of each iteration are depicted in Fig. 3. Although the initialization phase assessed only a limited number of structures with high selectivity and working capacity, the subsequent recommendations uncovered numerous structures in the upper right region of Fig. 3b–d. The minimum KPIs did not show impressive improvement due to the large deviations of the KPIs and the large amount of structures in the low-value region. Unlike methane storage, the model recommendations enhanced the mean and maximum KPIs. It is crucial to note that our model's objective is to efficiently populate regions of interest rather than exhaustively discover all top-performing candidates in the database. Therefore, not all the grey points in the upper-right space of the CO_2_/N_2_ selectivity—CO_2_ working capacity figures are recommended.

### 3.3 MOF recommendations based on band gaps

To demonstrate our recommendation model's effectiveness beyond gas adsorption and separation, we leveraged the QMOF database^15,56^ to study MOF electric properties. Specifically, we focused on recommending MOFs with band gaps falling within a specified range within the QMOF database, namely between 1 to 3 eV. MOFs with band gaps within this range exhibit semiconductor behavior and hold promise for applications such as photovoltaic devices, microelectronics, and sensors.^57,58^

**Fig. 4:**
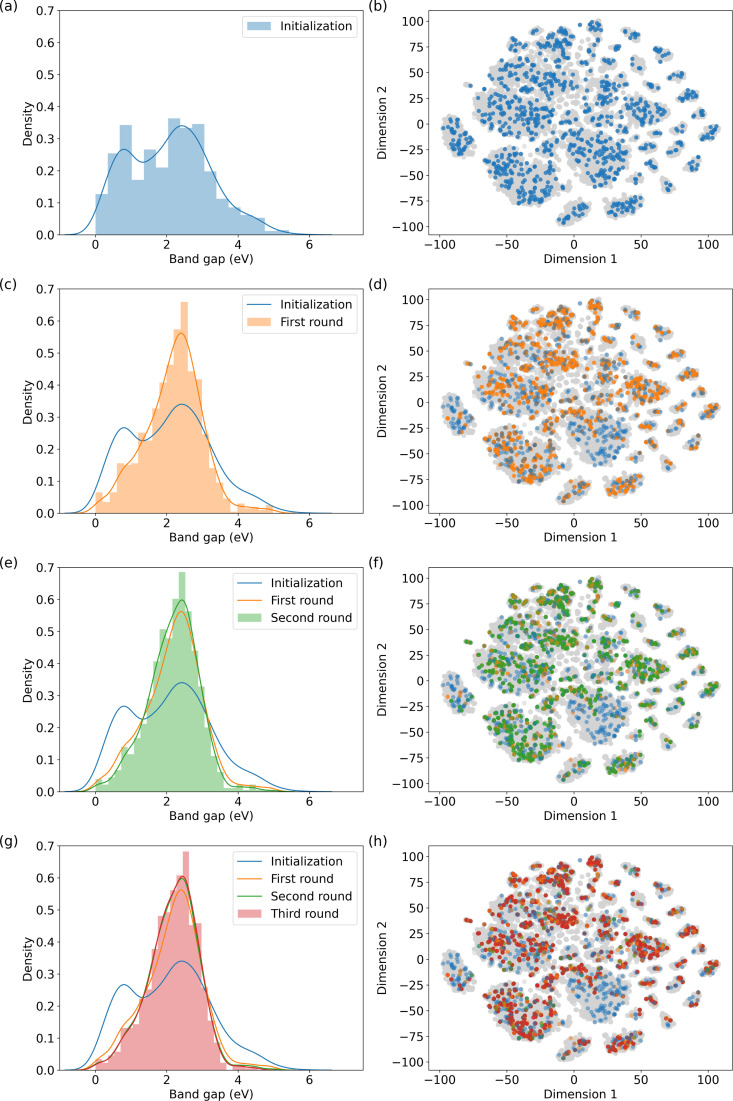
MOF recommendations in QMOF database based on the specified band gap range. (a), (c), (e), and (g) depict the normalized distribution of band gaps of the selected MOFs in each round; and (b), (d), (f), and (h) show their positions in the chemical space, respectively. The distributions of the recommendation rounds show a peak in the specified band gap range, indicating the success of the model recommendation in locating MOFs similar to those of the candidates from the previous round.

A similar recommendation procedure as described in Sections 3.1 and 3.2 is followed. The only difference lies in the material evaluation methods. Unlike performing molecular simulations in methane storage and carbon capture, we took advantage of the DFT-simulated band gaps from the QMOF database. The results of our recommendation process are illustrated in Fig. 4.

The results indicate that the model embeddings effectively capture quantum information, thereby enabling the identification of MOFs with comparable electronic properties. In Fig. 4, the left panel depicts the normalized distributions of MOF subsets across the initialization and subsequent three recommendation rounds. Initially, a discernible valley exists within the band gap range of 1 to 2 eV. However, this valley is filled through the recommendation rounds, indicating an augmentation in the number of MOFs falling within the specified band gap range. Furthermore, the number of MOFs with band gaps outside the queried range decreases with each round. We also mapped the MOFs in the QMOF database into two-dimensional space using t-SNE^59^ as shown on the right column of Fig. 4. Recommended MOFs from each round are highlighted. We stopped the recommendation iteration at the third round when the suggested subset closely aligns with the highlighted structures in Fig. 4f.

## 4 Discussion

### 4.1 MOF recommendations for methane storage and carbon capture

We further compared the top-performing MOFs identified by our recommendation model and those ranked by simulations. The model recommendations effectively cover a substantial portion (44% for methane storage and 61% for carbon capture) of around 100 top-performing MOFs in the dataset. This is achieved by evaluating less than 15% of the database with more than 22 000 MOFs, including initialization and three rounds of recommendations. This enhances the efficiency of identifying candidates for specific applications. To achieve a similar percentage mentioned above *via* random selection, one must evaluate at least 45% of the database (details elucidated in Section S3).†

**Fig. 5:**
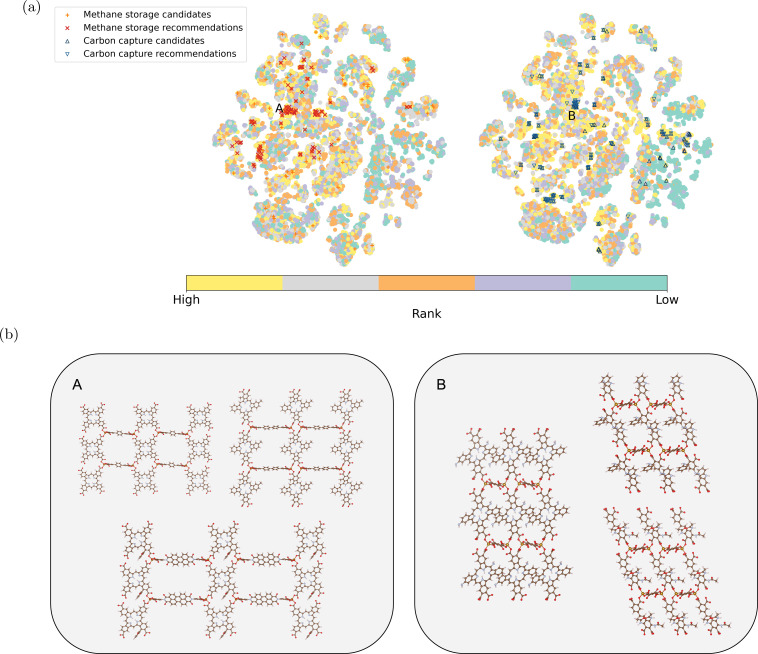
(a) Two-dimensional projection of the learned MOF embeddings using t-SNE.^59^ The color scheme represents the ranking of each material from the simulation for methane storage and carbon capture, respectively. The top 100 candidates for methane storage and carbon capture from simulation and model recommendations are highlighted. Candidates from simulations and model recommendations highly align in two regions labeled A and B. (b) Example MOFs are shown for Region A (methane storage) and Region B (carbon capture). These structures share the same topology and metal node, with variations in organic ligands. (The color scheme for the elements in the example structures follows: 
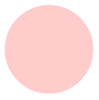
: H; 
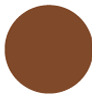
: C; 
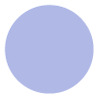
: N; 
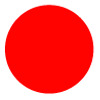
: O; 
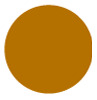
: Fe).

The model recommendations closely align with the top-performing MOFs from simulations, especially for carbon capture. This alignment is evident within regions labeled as A and B in Fig. 5a (for methane storage and carbon capture, respectively). Fig. 5b provides example MOFs from these regions. They share the same metal nodes and topology but exhibit versatile organic ligands. The diversity in organic ligands offers more options for MOF synthesis.^60–63^ Notably, the organic ligands for methane storage are generally longer, indicating larger pore sizes.^62^ Some candidates for methane storage remain undiscovered by our recommendation model as they are distributed across the chemical space. Moreover, the embedding vectors exhibit strong performance in downstream supervised regression models (see Section S4).†

### 4.2 Semantic analysis of MOF descriptors

During the training of a Doc2Vec model, the numeric word vectors undergo updates concurrently with the training of numeric document vectors, which encapsulate the content of a document. Similarly, the MOF vector learning process involves updating the embedding vectors associated with words based on their contextual surroundings. For instance, a pore diameter bin with larger values and a density bin with smaller values tend to appear in similar contexts, indicating shared semantic attributes and resulting in close proximity within the embedding space. The word corpus in this study includes MOF substructures and descriptors as shown in Fig. 1a. The learned embedding vectors offer valuable insights into the interrelations among various MOF characteristics by assessing their similarities.

**Fig. 6:**
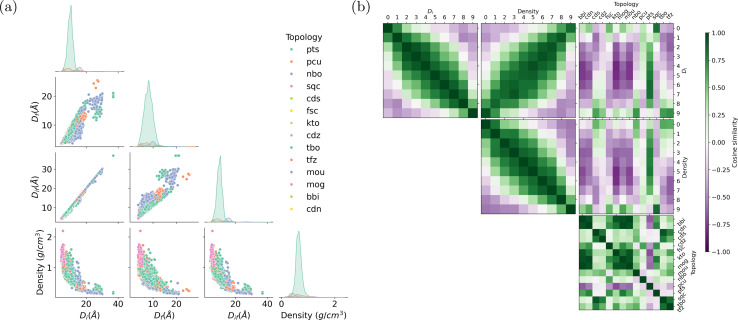
(a) Distribution of descriptors in the MOF documents of ARC-MOF database grouped by topology. Each point in the scatter plots represents a MOF. *D*_*i*_, *D*_*f*_, and *D*_*if*_ denote the largest included sphere, the largest free sphere, and the largest included sphere along the free sphere path, respectively. The most frequent topologies in this database are **pts**, **pcu**, and **nbo**. (b) The pairwise cosine similarity between pore diameter bins, density bins, and topologies. Vectors representing large pore sizes share high similarity with those representing low densities. High cosine scores between **nbo** and large pore-size bins indicate the promising potential of **nbo** in the design of MOFs with ultrahigh porosity.

We begin by presenting the statistical appearance frequency of descriptors within the MOF documents sourced from the ARC-MOF database, as illustrated in Fig. 6a. The diagonal axis of Fig. 6a reveals the distribution patterns of geometric descriptors for each topology. Descriptors such as **pts**, **pcu**, and **nbo**, representing distinct MOF topologies, exhibit the highest occurrence in the documents. Furthermore, the peaks associated with these descriptors shift from smaller to larger pore diameters. The three types of pore diameters (the largest included sphere, the largest free sphere, and the largest included sphere along the free sphere path) show strong positive correlations. In contrast, the pore diameter is negatively correlated with density, as we can see from the scatter plots in the last row of Fig. 6a.

Fig. 6b shows the pairwise cosine similarities among descriptors. Numeric descriptors, including pore diameters and densities, are categorized into ten bins ranging from small to large values. As expected, adjacent pore diameter and density bins exhibit cosine similarities close to 1, while bins that are farther apart tend to have cosine similarities close to −1. The off-diagonal line in the heatmap between pore diameter and density bins (the middle figure in the first row of Fig. 6b) highlights a darker green shade, indicating a negative correlation—larger pore diameters correspond to lower densities. Interestingly, some topologies exhibit distinct affinities with particular pore size ranges. For example, **nbo** exhibits high cosine similarity with large pore diameter bins, while **sqc** and **pcu** are closely associated with small pore diameter bins. The **pts** topology demonstrates similarity across all pore diameter ranges, particularly scoring high with medium pore sizes. These trends are consistent with the distributions shown in Fig. 6a. Cubic-shaped, straight and intersecting in a grid pattern, and hexagonal-shaped channels, respectively, characterize the **nbo**, **pcu**, and **pts** topologies. Each topology's distinctive geometric characteristics influence the MOFs' spatial constraints, resulting in preferences for specific pore sizes.^1,64,65^ Furthermore, the other topologies like **bbi**, **cdn**, and **fsc**, do not show a clear, obvious tendency toward pore diameters or densities due to their limited appearance in the document corpus or their weak correlation with porosity. The control of topology is critical in tuning the porosity of MOFs, a process significantly influenced by the ligand functionalization and synthesis conditions.^66^

In addition to semantic analysis between geometric descriptors, we extend our exploration to assess the similarity among molecular fragments (see Section S5).† This comprehensive analysis enhances our understanding of the intrinsic relationships between MOF functional groups, porosity, topology, *etc.*

## 5 Conclusion

In this work, we presented a deep-learning-based recommendation system for metal–organic frameworks (MOFs), employing an unsupervised model built on Doc2Vec. The iterative recommendation system can be a valuable tool for exploring the vast MOF chemical space, aiding researchers in identifying potential MOFs for tailored applications without prior knowledge about the databases. We demonstrate that it is a practical and resource-effective approach for specific applications through methane storage and carbon capture and its success in capturing MOFs' quantum properties. Beyond recommendations, the model unveils the interrelations of various MOF characteristics and provides insights into materials design. In an era dominated by large language models, our work showcases a novel application of lightweight language models in materials discovery.

## 6 Methods

### 6.1 Molecular simulation

The gas adsorption and separation performance of MOFs is evaluated by molecular simulation. We applied the TraPPE force-fields^67^ to describe CH_4_, CO_2_, and N_2_ molecules. Lennard-Jones 12-6 potential using UFF parameters^68^ were used to simulate the gas-framework interactions, truncated at 12.8 Å with tail corrections.^69^ Electrostatic interactions were modeled with Ewald summation. All the molecular simulations were performed with RASPA.^70^ The Henry coefficients of gas molecules at 298 K are simulated by Widom insertions. Grand-canonical Monte Carlo (GCMC) simulations with 6000 equilibrium cycles followed by 6000 production cycles were used to simulate the gas uptakes. We simulated methane adsorption and desorption at 298 K at 65 and 5.8 bar, respectively. The mixture gas for carbon capture contains 15% CO_2_ and 85% N_2_. The CO_2_ and N_2_ adsorption were simulated at 298 K with an external pressure of 1 bar and desorption conditions of 363 K and 0.1 bar. The deliverable capacity (methane storage) and working capacity (carbon capture) were computed by the difference in loadings at adsorption and desorption conditions.

### 6.2 Crystallographic information encoding

As we adopt a natural language processing (NLP) framework, the geometric and structural information of the MOFs are encoded into textual documents. Each document is assigned a unique title. The encoding of chemistry structure utilizes the Weisfeiler-Lehman (WL) kernel algorithm like the work of Narayanan *et al.*^71^

The WL algorithm mines through the subgraphs of graphs to compare how similar two graphs are. We applied a similar strategy: considering a MOF as a graph with nodes representing atoms and edges representing bonds. Firstly, we label the atoms in the MOFs according to their species. Each atom's first-order neighbors are extracted, called the substructures. Next, we label the substructures with the sorted atom species. We repeat the labeling process to the second-order neighbors and retain the unique labels in each step.

Additionally, we categorize continuous descriptors into ten bins to encode geometric information. To be independent of probes, we only apply the intrinsic geometric characteristics of MOFs, including density, the largest included sphere diameter, the largest free sphere diameter, and the largest included sphere diameter along the free sphere path. We included all three types of pore diameters since a larger corpus typically yields better embeddings, especially when the words are not rare. Binning the KPI values enables the discretization of continuous data into distinct categories. Categorial descriptors like topology are also appended to the documents.

### 6.3 Doc2Vec model

We employ the gensim^72^ package to implement the Doc2Vec model using the distributed memory algorithm, which can capture the context of the MOF fragments. This algorithm simultaneously learns reliable embeddings for MOFs and document words, facilitating further analysis. We embed the MOF documents and associated words into 1000-dimensional continuous vectors. The maximum distance between the current and predicted words within a document was set to 100. No word is dropped due to its scarcity in the corpus. The learning rate was initialized at 3 × 10^−2^ and gradually reduced to at least 1 × 10^−5^ throughout 100 training epochs.

## Data availability

The recommendation framework is available at https://github.com/XiaoqZhang/mofgraph2vec.git as open source. The datasets and trained models are available at Zenodo at https://zenodo.org/doi/10.5281/zenodo.11045846.

## Author contributions

X. Z. developed the recommendation system and analyzed the results. K. M. J. proposed the idea of the project and contributed to discussions. B. S. led the project and provided directions. All authors contributed to the manuscript and have approved the final version of the manuscript.

## Conflicts of interest

There are no conflicts to declare.

## Supplementary Material

DD-003-D4DD00116H-s001
