# Supplementary material for: Deep learning-based recommendation system for metal–organic frameworks (MOFs)
Source: Digit Discov. 2024 Jun 10;3(7):1410–20. doi: 10.1039/d4dd00116h (PMC11235176; doi:10.1039/d4dd00116h)
Supplement: DD-003-D4DD00116H-s001 [file DD-003-D4DD00116H-s001.pdf]

Electronic Supplementary Material (ESI) for Digital Discovery.
This journal is © The Royal Society of Chemistry 2024

Deep Learning-Based Recommendation System for Metal-Organic Frameworks (MOFs)

Xiaoqi Zhang [1], Kevin Maik Jablonka [1,2,3], and Berend Smit [*1]

[1]Laboratory of Molecular Simulation (LSMO), Institut des Sciences et Ingénierie Chimiques, Ecole Polytechnique Fédérale de Lausanne(EPFL), Rue de l'Industrie 17, CH-1951 Sion, Valais, Switzerland

[2]Laboratory of Organic and Macromolecular Chemistry (IOMC), Friedrich Schiller University Jena, Humboldtstrasse 10, 07743 Jena, Germany.

[3]Helmholtz Institute for Polymers in Energy Applications Jena (HIPOLE Jena), Lessingstrasse 12-14, 07743 Jena, Germany

S1 Statistical analysis of cosine similarity

The model recommendation is based on the cosine similarity of embedding vectors as described in Section 2. To verify the effectiveness of the model recommendation, the fundamental question one would like to address is: what is the threshold value to assess the recommendation similarity? Explicitly, which thresholds are suggested for MOFs with similarity scores? Hence, we conducted a statistical survey of the similarity score of the relevant subsets, for which the results are shown in Figure S1. We computed the probability of observing the cosine scores of each MOF subset above a threshold value. For the same probability, the threshold becomes larger from initialization to the subsequent recommendation rounds, indicating the structures in the recommendation rounds share significant similarities. Besides, the probability drops down more rapidly in the QMOF database than in the ARC-MOF database in the initialization stage, indicating that finding a similarity value exceeding a threshold value is more probable to be a rare event. This reflects the structure similarity in the overall database, which can be used to evaluate the diversity of the database.

We also analyzed the distribution of the similarity score in each round, as shown in the first row

*berend.smit@epfl.ch

of Figure S1. In the context of methane storage, the distributions for the three recommendation rounds are similar, with two peaks around 0.7 and 1.0, especially for the second and third rounds. For carbon capture, the density of high cosine similarity is extremely large. When suggesting MOFs with band gaps within a specific range, the cosine similarity spans over a large range. Compared to methane storage and carbon capture, the similarity distribution for the band gap is generally wider due to the diversity of the QMOF database.

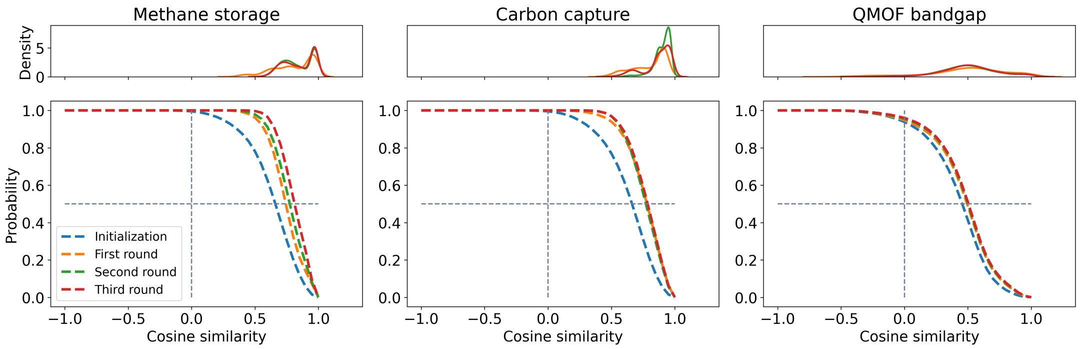

Supplementary Figure S1: Statistical analysis of cosine scores in the ARC-MOF (methane storage and carbon capture) and QMOF (QMOF band gap) databases. The first row shows the normalized distributions. The second row depicts the probability of observing the cosine scores of an MOF subset above a threshold value.

S2 Top 100 recommendations in simulations

As mentioned in Section 4.1, 44% for methane storage and 61% for carbon capture of the top 100 recommended materials also appear in the 100 top-performing materials ranked by the simulations. We show the rankings of the 100 recommendations in simulations in Figure S2. The recommendations are within the top 300 for methane storage and within the top 200 for carbon capture.

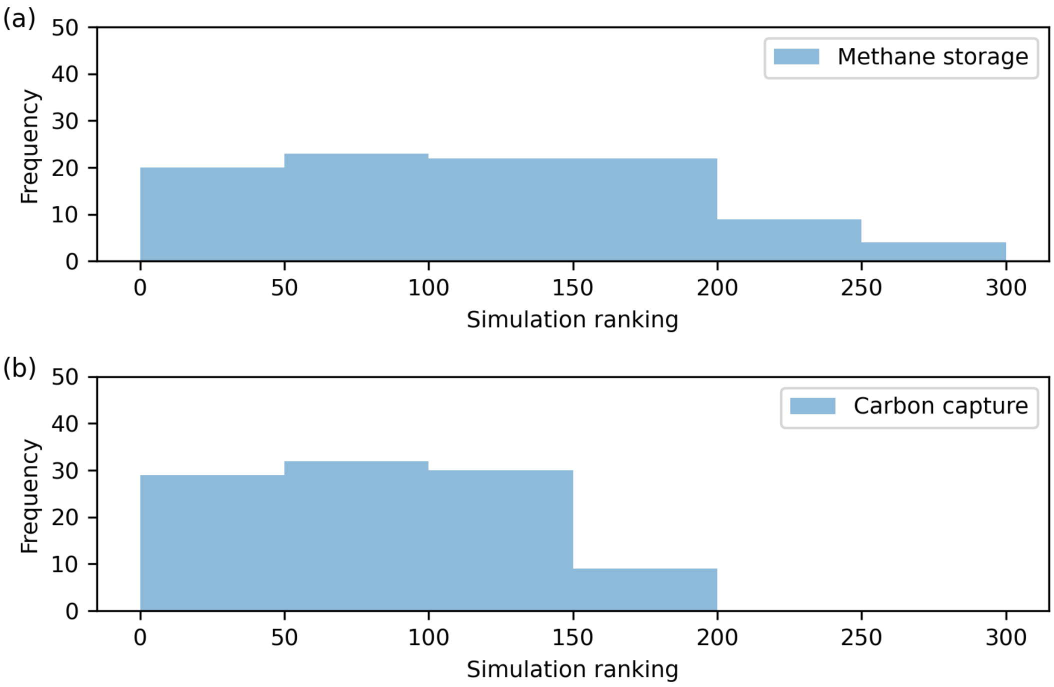

Supplementary Figure S2: The top 100 recommendations for (a) methane storage and (b) carbon capture in simulation rankings. All the recommendations are within the top 300 materials ranked by simulations.

S3 Comparison with random selections

The probability of finding k candidates among the K top-performing ones in the n MOFs drawn can be calculated by

$$P(X = k) = \frac{\binom{K}{k} \cdot \binom{N-K}{n-k}}{\binom{N}{n}} \tag{S1}$$

Where:

- N is the number of MOFs in the ARC-MOF database we used in this work (22,035)
- K is the number of candidates identified through molecular simulations. Here, we set it to 100
- n is the number of MOFs drawn by random selection
- k is the number of candidates covered in the draw

We plot the probability distribution as a function of the percentage of identified candidates in Figure S3. To reach a comparable percentage with our model (around 40%–60%), one needs to

evaluate at least 10,000 MOFs (around 45% of the database).

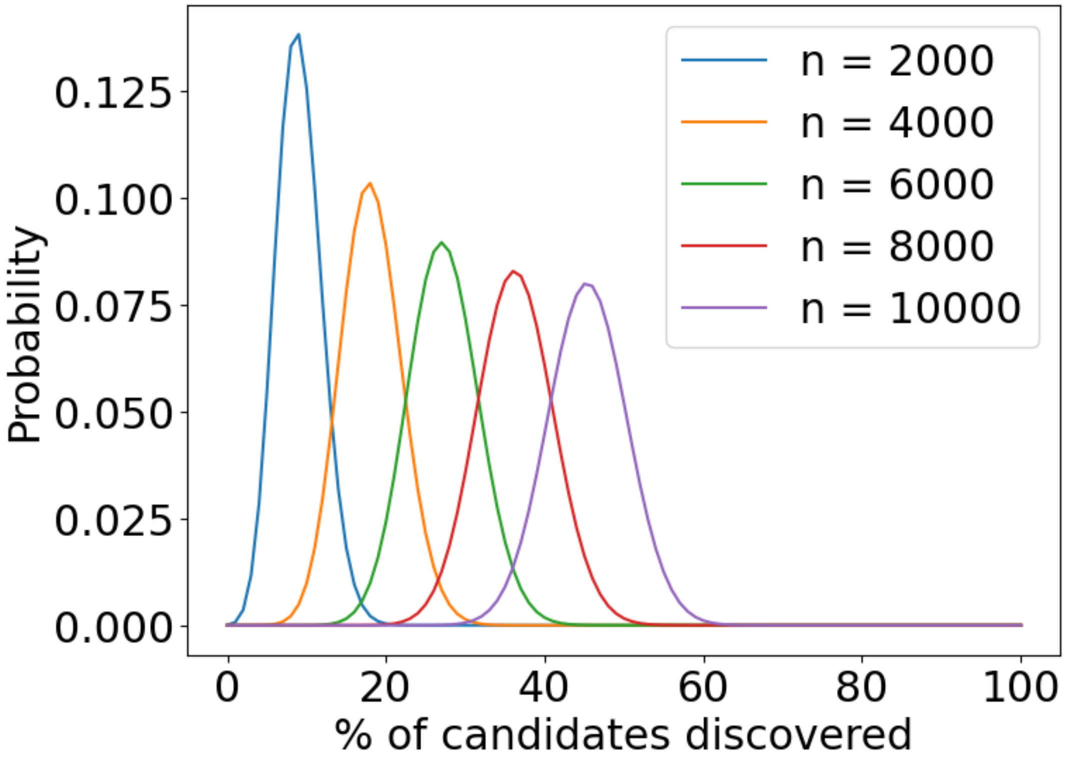

Supplementary Figure S3: The probability distribution of identifying candidates in a randomly drawn MOF subset.

S4 Doc2Vec embeddings in supervised tasks

The embeddings from the Doc2Vec model can also be used as MOF feature vectors for supervised models. We display regression model performances in predicting the KPIs for methane storage (Henry coefficient in logarithm and CH_4 deliverable capacity) and carbon capture (CO_2/N_2 selectivity and CO_2 working capacity) in Figure S4. We used the extreme gradient boosting (XGBoost) algorithm[1] to train the regression model, splitting the data into training and testing sets with a ratio of 80% to 20%. The training and testing sets were stratified based on the target values. Hyperparameters were fine-tuned through a 5-fold grid search cross-validation using the training dataset. Overall, the model predictions perform well. However, some extreme values in CO_2/N_2 selectivity make the training extremely challenging.

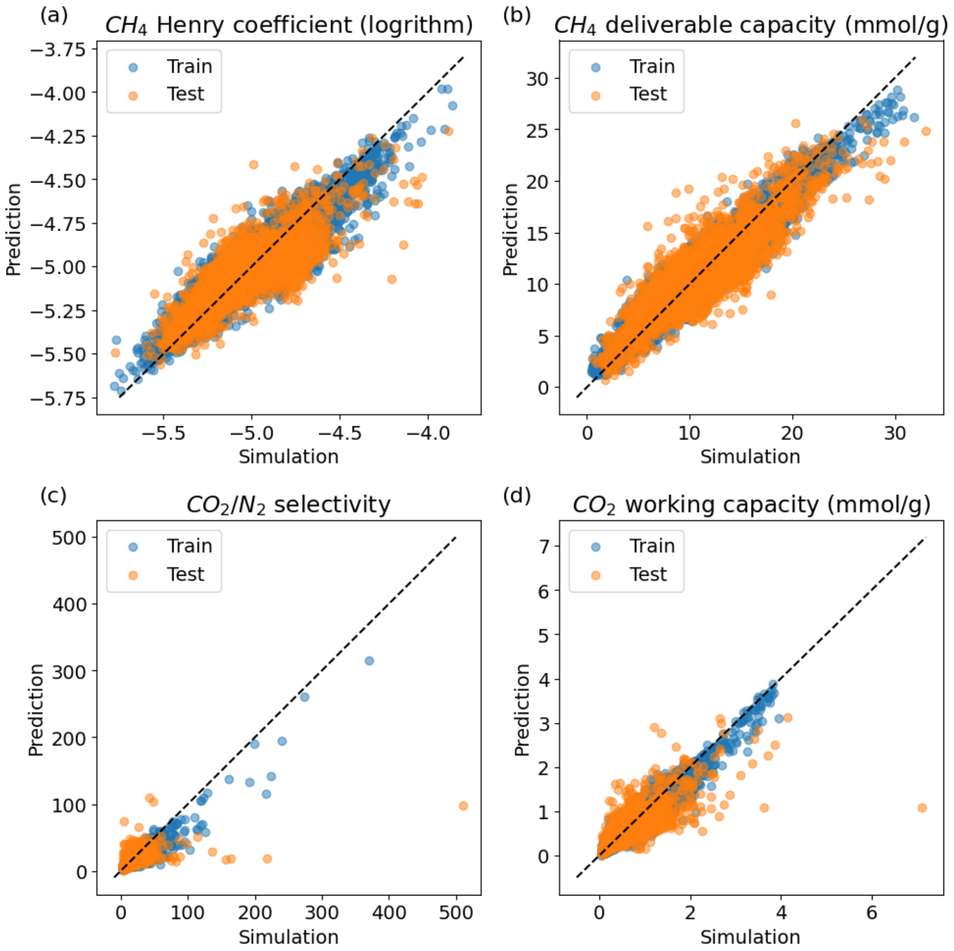

Supplementary Figure S4: Parity plot for the supervised regression model. Embedding vectors from the recommendation model can be utilized in supervised models, performing well for methane storage. Specifically, these models achieve an R^2 score of 0.60 for the Henry coefficient and 0.76 for deliverable capacity.

S5 Semantic analysis of molecular fragments

We display the similarity confusion matrix of some molecular fragments in Figure S5. They are often the functional groups of organic linkers. As expected, halogen atoms share high similarity scores.

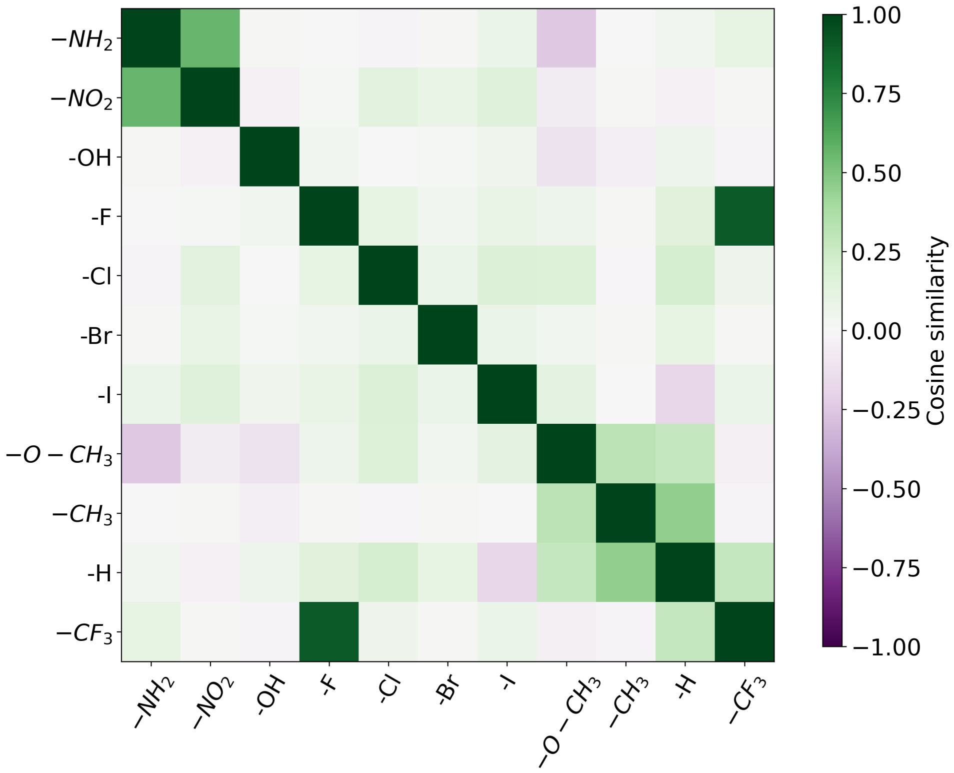

Supplementary Figure S5: Cosine similarity matrix of molecular fragments.

References

[1] Chen, T.; Guestrin, C. XGBoost: A Scalable Tree Boosting System. Proceedings of the 22nd ACM SIGKDD International Conference on Knowledge Discovery and Data Mining. New York, NY, USA, 2016; pp 785–794.
